# Clade 8 and Clade 6 Strains of *Escherichia coli* O157:H7 from Cattle in Argentina have Hypervirulent-Like Phenotypes

**DOI:** 10.1371/journal.pone.0127710

**Published:** 2015-06-01

**Authors:** Natalia Amigo, Elsa Mercado, Adriana Bentancor, Pallavi Singh, Daniel Vilte, Elisabeth Gerhardt, Elsa Zotta, Cristina Ibarra, Shannon D. Manning, Mariano Larzábal, Angel Cataldi

**Affiliations:** 1 Instituto de Biotecnología, CICVyA, Instituto Nacional de Tecnología Agropecuaria, Hurlingham, Argentina; 2 Instituto de Patobiologia, CICVyA, Instituto Nacional de Tecnología Agropecuaria, Hurlingham, Argentina; 3 Microbiología, Facultad de Ciencias Veterinarias, Universidad de Buenos Aires, Buenos Aires, Argentina; 4 Departamento de Fisiología, IFIBIO-CONICET, Facultad de Medicina, Universidad de Buenos Aires, Buenos Aires, Argentina; 5 Department of Microbiology and Molecular Genetics, Michigan State University, East Lansing, Michigan, United States of America; USDA-ARS-ERRC, UNITED STATES

## Abstract

The hemolytic uremic syndrome (HUS) whose main causative agent is enterohemorrhagic *Escherichia coli* (EHEC) O157:H7 is a disease that mainly affects children under 5 years of age. Argentina is the country with the highest incidence of HUS in the world. Cattle are a major reservoir and source of infection with *E*. *coli* O157:H7. To date, the epidemiological factors that contribute to its prevalence are poorly understood. Single nucleotide polymorphism (SNP) typing has helped to define nine *E*. *coli* O157:H7 clades and the clade 8 strains were associated with most of the cases of severe disease. In this study, eight randomly selected isolates of EHEC O157:H7 from cattle in Argentina were studied as well as two human isolates. Four of them were classified as clade 8 through the screening for 23 SNPs; the two human isolates grouped in this clade as well, while two strains were closely related to strains representing clade 6. To assess the pathogenicity of these strains, we assayed correlates of virulence. Shiga toxin production was determined by an ELISA kit. Four strains were high producers and one of these strains that belonged to a novel genotype showed high verocytotoxic activity in cultured cells. Also, these clade 8 and 6 strains showed high RBC lysis and adherence to epithelial cells. One of the clade 6 strains showed stronger inhibition of normal water absorption than *E*. *coli* O157:H7 EDL933 in human colonic explants. In addition, two of the strains showing high levels of Stx2 production and RBC lysis activity were associated with lethality and uremia in a mouse model. Consequently, circulation of such strains in cattle may partially contribute to the high incidence of HUS in Argentina.

## Introduction

*Escherichia coli* O157:H7 is a globally important zoonotic pathogen capable of causing hemorrhagic colitis and hemolytic uremic syndrome (HUS) in humans. HUS is widely distributed in the world and is described as an epidemy of low incidence rate in industrialized countries (1 to 3 cases per 100,000 children aged under 5 years) [1,2]. However, in Argentina, a country with the highest HUS incidence in the world [3,4], 12 to 14 cases were reported per 100,000 children aged under 5 years of age with about 400 new cases reported annually in the last decade. In Argentina, HUS is the leading cause of acute kidney failure in children and the second cause of chronic renal failure, and is also responsible for 20% of kidney transplants in children and adolescents [5].

A high percentage of domestic cattle in many countries are colonized by Shiga toxin-producing *E*. *coli* (STEC), which do not cause illness in cattle. Enterohemorrhagic *E*. *coli* (EHEC) [6,7,8,9] are STEC and can be differentiated by the presence of the 35.6 kb pathogenicity island called the locus of enterocyte effacement (LEE). This pathogenicity island encodes for a Type Three Secretion System (T3SS) [10,11] and gives strains the ability to produce a histopathological lesion in the intestinal epithelium known as an attaching and effacing (A/E) lesion. A/E lesions are characterized by the close adherence to enterocytes and depletion of intestinal microvilli.

The production of Shiga toxin (Stx), also known as verocytotoxin (VT), represents the most important virulence attribute in EHEC. After colonization, Stx is produced and released into the intestine. Most strains produce at least one Stx variant, which may be of type 1 (Stx1) and/or type 2 (Stx2) [12].

Isolates of EHEC O157:H7 have been shown to be genotypically diverse by different methods, including PFGE [13], octomer-based genome scanning [14], and multilocus variable number of tandem repeats analysis [13]. Variations in disease severity between outbreaks caused by *E*. *coli* O157:H7 are evident and depend on specific genotypes [15,16,17]. For example, the 1993 multistate outbreak in North America [18] and the 1996 outbreak in Sakai, Japan [19] had low rates of hospitalization and HUS [20]. In comparison, two outbreaks in the United States caused by contaminated lettuce and spinach had much higher frequencies of both hospitalization (mean, 63%) and HUS (mean, 13%) [21]. These outbreak strains along with more than 500 additional strains have been previously characterized by the analysis of 96 SNPs. By phylogenetic analyses, Manning et al [15] identified 39 SNP genotypes in this broad collection of *E*. *coli* O157 isolates, which differed at 20% of the SNP loci and were separated into nine different clades. The *stx* profiles varied among strains belonging to different clades and certain clades were associated with clinical symptoms. HUS patients, for example, were significantly more likely to be infected with strains of clade 8, which had increased in frequency over a 5-year period in Michigan [15]. Accordingly, it has been suggested that a subpopulation or more virulent lineage has emerged. These clade 8 strains have been identified in various clinical cases on multiple continents and countries, including Argentina, since 1984 [15]. To date, those factors that are important for enhancing the virulence of this subset of strains are not completely understood. The clade 8 strains have higher Stx2 expression than other clades [22] and have unique genetic features that may be important for the disease [23].

We and other groups have previously identified strains belonging to clade 8 in cattle from different provinces of Argentina [24,25]. For this reason, we sought to more closely examine the genetic relationships between strains by screening for previously described SNPs [15] and assessing their pathogenic potential following exposure to epithelial cells and infection in mice.

## Materials and Methods

### Bacterial isolates

**Fig 1:**
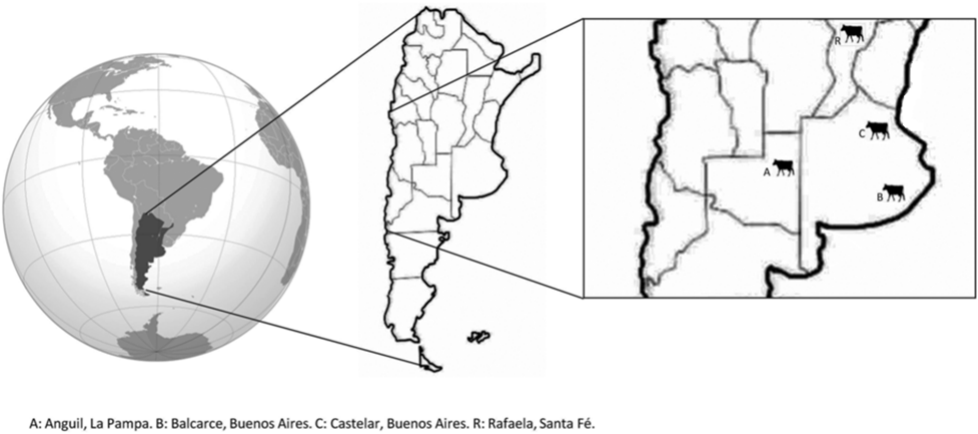
Map showing the geographical location of cattle where *Escherichia coli* O157:H7 isolates were taken. A: Anguil, La Pampa. B: Balcarce, Buenos Aires. C: Castelar, Buenos Aires. R: Rafaela, Santa Fe.

Eight *E*. *coli* O157:H7 isolates recovered from cattle in the central humid Pampa region of Argentina from 2002 to 2011 (Fig 1 and Table 1) were examined along with 2 human local isolates from HUS cases. The cattle isolates were collected by rectal swab and obtained from private or INTA farms with permission from the farmer or manager. The standard *E*. *coli* O157:H7 EDL933 strain recovered from a patient in USA and the nonpathogenic *E*. *coli* DH5α were included in the study as positive and negative control, respectively, in virulence assays. Bacteria were grown aerobically on Luria-Bertani (LB, Difco Laboratories, USA) agar plates or in LB broth at 37°C. For functional studies, bacterial strains were grown in LB broth overnight at 150 rpm and then diluted 1/50 in Dulbecco’s modified Eagle’s medium/(DMEM)-F12 medium and grown to exponential phase (optical density (OD) at 600 nm of 0.3–0.4) at 37°C at 50 rpm.

**Table 1:** Epidemiological, and genotypic characteristic of *E*. *coli* O157:H7 strains.

Strain	Host	Location	Origin	Isolation year	Stx1	Stx2	Stx2 subtype	Clade as in [28] (nts SNP 539, 1060, 438, 630)^2^	Clade x 32^3^	Stx2 integration loci
EDL933	Human,	patient	USA		+	+	*stx2a*	3 (C-G-C-T)	clade 3	*wrbA*
146N	Bovine	abattoir	ARG		+	+	*stx2c*	3(C-G-C-T)	ND	*yehV*
438/99	Bovine	abattoir	ARG	1999	-	+	*stx2c*	3(C-G-C-T)	ND	ND*
7.1 Anguil	Bovine	farm^1^	ARG	2009	-	+	*stx2a-stx2c*	none (C-T-C-T)	clade 6	
9.1 Anguil	Bovine	farm^1^	ARG	2009	-	+	*stx2a-stx2c*	none(A-T-C-T)	clade 8	*yehV*
Vac 07–1	Bovine	farm^1^	ARG	2007	-	+	*stx2a-stx2c*	none-(A-G-C-T)	clade 8	
125/99	Human	patient	ARG		-	+	*stx2a*	none- (A-G-C-T)	clade 8	*yehV*
Rafaela II- 827	Bovine	farm^1^	ARG	2009	-	+	*stx2a-stx2c*	none-(A-G-C-T)	clade 8	ND*
Balcarce 14.2	Bovine	farm^1^	ARG	2009	-	+	*stx2a-stx2c*	none- (A-G-C-T)	clade 6	*yehV*
Balcarce 24.2	Bovine	farm^1^	ARG	2009	-	+	*stx2a-stx2c*	none- (A-G-C-T)	clade 8	
Neuquén 7562	Human	patient	ARG	2003	-	+	*stx2c*	none-(A-G-C-T)	clade 8	*yehV*

1 For geographical location of farms see Fig 1

2 nucleotide substitution in the four SNPs descrbed in [28]

3. clades as determined using the 32 SNP method described here

### Genotyping

Stx1 and Stx2 were detected using primers described by Olsvik and Strockbine [26] (Table 2). Subtyping to discriminate Stx2 variants was performed by PCR by using primers described by Scheutz et al [27] in Table 2.

**Table 2:** Primers used in this study.

Primer	Sequence	Purpose	Tm (C°)	amplicon size (bp)	References
Vt1-a	CAGTTAATGTGGTGGCGAAG	Stx1 detection	55	894	[26]
Vt1-b	CTGCTAATAGTTCTGCGCATC	Stx1 detection	55	894	[26]
Vt2-a	CTTCGGTATCCTATTCCCGG	Stx2 detection	45	478	[26]
Vt2-b	GGATGCATCTCTGGTCATTG	Stx2 detection	45	478	[26]
stx2a-F2	GCGATACTG**R**G**B**ACTGTGGCC	Stx2 variant confirmedstx2a	66	349 347	[27]
stx2a-R3	CCG**K**CAACCTTCACTGTAAATGTG	Stx2 variant confirmedstx2a	66	349 347	[27]
stx2a-R2	GCCACCTTCACTGTGAATGTG	Stx2 variant confirmedstx2a	66	349 347	[27]
stx2b-F1	AAATATGAAGAAGATATTTGTAGCGGC	Stx2 variant confirmedstx2b	65	251	[27]
stx2b-R1	CAGCAAATCCTGAACCTGACG	Stx2 variant confirmedstx2b	65	251	[27]
stx2c-F1	GAAAGTCACAGTTTTTATATACAACGGGTA	Stx2 variant confirmed stx2c	65	177	[27]
stx2c-R2	CCGGCCAC**Y**TTTACTGTGAATGTA	Stx2 variant confirmed stx2c	65	177	[27]
stx2d-F1	AAARTCACAGTCTTTATATACAACGGGTG	Stx2 variant confirmedstx2d	65	179 235 280	[27]
stx2d-R1	TT**Y**CCGGCCACTTTTACTGTG	Stx2 variant confirmedstx2d	65	179 235 280	[27]
stx2d-O55-R	TCAACCGAGCACTTTGCAGTAG	Stx2 variant confirmedstx2d	65	179 235 280	[27]
stx2d-R2	GCCTGATGCACAGGTACTGGAC	Stx2 variant confirmedstx2d	65	179 235 280	[27]
stx2e-F1	CGGAGTATCGGGGAGAGGC	Stx2 variant confirmedstx2e	66	411	[27]
stx2e-R2	CTTCCTGACACCTTCACAGTAAAGGT	Stx2 variant confirmedstx2e	66	411	[27]
stx2f-F1	TGGGCGTCATTCACTGGTTG	Stx2 variant confirmedstx2e	66	411	[27]
stx2f-R1	TAATGGCCGCCCTGTCTCC	Stx2 variant confirmedstx2e	66	411	[27]
stx2g-F1	CACCGGGTAGTTATATTTCTGTGGATATC	Stx2 variant detectionstx2g	62	573	[27]
stx2g-R1	GATGGCAATTCAGAATAACCGCT	Stx2 variant detectionstx2g	62	573	[27]
ECs2357f	TTAACCTTTTGTGGCGAACC	SNP ECs2357 detection	58	253	This study
ECs2357r	TACGGTTTGCCGCAGTTATT	SNP ECs2357 detection	58	253	This study
ECs2521f	CCGTAGCAGGTTCGGTAAAA	SNP ECs2521 detection	60	200	This study
ECs2521r	CGGTTCCAGTTCGTCGATAA	SNP ECs2521 detection	60	200	This study
ECs3881**f**	GAGAACGGCTACGCGTACAT	SNP ECs2521 detection	60	200	This study
ECs3881**r**	CGTTCCACACCTTTCTGGTT	SNP ECs2521 detection	60	200	This study
ECs4130**f**	GGGCTGCTGATTTTTGGTAT	SNP ECs4130 detection	58	229	This study
ECs4130**r**	CAGGCGACAGAATATCGTCA	SNP ECs4130 detection	58	229	This study
*sbcB*-F	GACAGCAGAAACAACGGATTTAAC	*sbcB* insertion site	59	406	[33]
*sbcB*-R2	TCCAGGCGTAAGGATCGTAG	*sbcB* insertion site	59	406	[33]
A	AAGTGGCGTTGCTTTGTGAT	*yehV* insertion site	60	340	[55]
B	AACAGATGTGTGGTGAGTGTCTG	*sbcB* insertion site	59	406	[33]
C	AGGAAGGTACGCATTTGACC	*wrbA* insertion site	60	314	[33]
D	CGAATCGCTACGGAATAGAGA	*wrbA* insertion site	60	314	[33]
*argW*-A	CCGTAACGACATGAGCAACAAG	*argW* insertion site	55	216	[33]
*argW*-D	AATTAGCCCTTAGGAGGGGC	*wrbA* insertion site	60	314	[33]

### SNP typing for clade determination

The primers in Table 2 were initially used to generate amplicons to identify SNPs and classify strains into clades according to the algorithm developed by Riordan et al [28]. In addition, an expanded set of 23 of the 32 previously identified SNPs capable of differentiating the original SNP genotypes [15] were evaluated using the GoldenGate genotyping assay (Illumina; San Diego, CA) [29]. SNP loci evaluated in this assay are highlighted in SNP_info (S1 Table). Sequences specific for a set of 30 strains were included as controls to represent the original SNP genotypes identified by Manning et al. [15]; clades were defined using these previously defined control strains. All 23 SNPs were concatenated in MEGA6 [30] and the evolutionary history was inferred using the Neighbor-Joining method [31]. The bootstrap test was used with 1,000 replicates to identify clusters, or clades [32] and evolutionary distances were presented as the number of SNP differences per site. Sequences specific for a set of nine strains were included as controls to represent the original SNP genotypes identified previously [15]. The insertion sites of *stx2* phages were determined according to Shringi et al [33] (Table 2).

### Shiga-Toxin detection Kit

The expression of Stx was determined in filtered culture supernatants, with the addition or absence of mitomycin C, by using RIDASCREEN Kit Verotoxin enzyme immunoassay (R-Biopharm Latin America). Its expression was semi-quantified according to Beutin et. al [34].

### Stx toxicity in Vero cells

Strains were incubated overnight at 37°C and 200 rpm in 5 ml of LB broth and subsequently the culture supernatant was obtained by centrifugation and filtration (0.22 μm filters). Filtered culture supernatants were assayed for cytotoxicity on Vero cells as previously described [35]. Briefly, Vero cell monolayers grown in 96-well plates were treated for 72 h under growth-arrested conditions (serum-free medium) with filtered culture supernatant from different strains. At the end of the incubation, plates were washed twice with PBS (145 mM NaCl, 10 mM NaH_2_PO_4_, pH 7.2) and incubated for 2 h with freshly diluted neutral red in PBS to a final concentration of 50 μg/ml. Cells were then washed with 1% CaCl_2_ and 4% formaldehyde twice and then were solubilized in 1% acetic acid and 50% ethanol. Absorbance at 546 nm was read in an automated plate spectrophotometer. Results were expressed as percent viability, with 100% represented by cells incubated under identical conditions but without treatment. The 50% cytotoxic dose (CD_50_) corresponded to the dilution required to kill 50% of Vero cells.

The results are the means of three experiments. The statistical difference was expressed as the P value determined by a two-way ANOVA and a Bonferroni post-test.

### Adherence to Caco-2, HeLa and HEp-2 cells

The ability of *E*. *coli* strains to adhere to HeLa (ATCC-CCL2), Caco-2 (ATCC-HTB-37) and HEp-2 (ATCC-CCL-23) cell monolayers was assessed. Sterile glass coverslips (12 mm) were inoculated with 10^5^ cells per well. The cells were grown to 90% confluence at 37°C in 5% CO_2_ in 24-well plates (Corning) in DMEM with 10% (vol/vol) heat-inactivated fetal bovine serum, 2 mM L-glutamine, penicillin (100,000 IU/liter), and streptomycin (100 mg/liter) [36]. Before use, the cells were washed with sterile phosphate-buffered saline (PBS; pH 7.4) and replenished with DMEM. The bacterial strains were grown in LB broth overnight at 37°C. For qualitative and quantitative assays, tissue culture cells were incubated with 10^7^ bacteria per well (MOI 100) for 5 h at 37°C. The monolayers were washed, fixed, and stained with Giemsa solution for microscopic evaluation. For *E*. *coli* adherence quantification, the infected monolayers were washed three times with PBS and the adherent bacteria were recovered with 200 ul of 0.1% Triton X 100 in PBS buffer and plated on LB agar plates. Data are expressed as the CFU from adhered bacteria from triplicate wells and are the mean of at least three separate experiments. The statistical difference was expressed as the P value determined by a Tukey's Multiple Comparison Test analysis.

### Red blood cell (RBC) lysis assay

The hemolytic activity exhibited by T3SS-encoding *E*. *coli* strains [37] was evaluated. Overnight grown cultures (1:100 dilution of EHEC O157:H7) were incubated with 5% suspension of red blood cells (RBC) in DMEM without phenol red for 4 h at 37°C under a 5% CO_2_ atmosphere as described by Larzábal, et al [38]. Briefly, the suspension was removed from the plates and centrifuged at 12,000g for 1 min. The supernatants were monitored for the presence of released hemoglobin by measuring OD_543_. The statistical difference was expressed as the P value determined by a Tukey's Multiple Comparison Test analysis. In order to eliminate any possible action of other virulence factors of *E*. *coli* strains on the RBCs lysis, such as secretory enterohemolysin (Ehly), or Shiga toxins (Stx), the bacteria were incubated in DMEM for 5 h to allow the secretion of these factors. The culture supernatant was filtered (0.22 micron filter) to have secretory virulence factors free of bacteria. The supernatant was incubated with a 5% suspension of RBCs in PBS at pH 7.4.

### Functional test using human intestinal tissue

Colon fragments used in this study were obtained from surgeries performed on three adult cancer patients (informed consent was obtained) at the “Servicio de Cirugía Gastroenterológica, Hospital Churruca-Visca”, Buenos Aires, Argentina. The Ethics Committee of the Universidad de Buenos Aires approved the use of human tissues for research purposes. The colonic mucosa was obtained and mounted as a diaphragm on a modified Ussing chamber as previously described [39]. Transepithelial net water flux (Jw) and electrical measurements were used as functional test. Jw was recorded automatically across an Ussing chamber connected to a special electro-optical device [40]. The sensitivity of this instrument is approximately 50 nl. The spontaneous potential difference (PD) was simultaneously recorded in the other chamber across the micro-reference electrodes (Harvard Apparatus Inc, USA) placed adjacent to the epithelium under open-circuit conditions. The short circuit current (Isc) was measured with an automatic voltage clamp system that kept the PD at 0 mV. The transepithelial resistance (Rt) was calculated from the Isc and open-circuit PD values, according to Ohm’s law. The parameters were stabilized and then 200 μl (approximately 10^8^ CFU) of bacterial culture of EDL933 or 7.1 Anguil were added to the mucosal side of each colon tissue (time 0). Then, both Jw and Isc were simultaneously recorded for 1 h. Because of tissue variability, data are analyzed as ∆Jw where ∆Jw = Jw (at a given time)—Jw (at time 0) and ∆Isc, where ∆Isc = Isc (at a given time)—Isc (at time 0). Each assay was carried out three times with colon fragments obtained from different patients. The results are reported as mean ± 1 standard error. Statistical significance between two mean values obtained for two experimental conditions were calculated using the "t" Student test. For statistical analysis of curves, ANOVA was used to evaluate changes in Jw of each of the strains according to the time.

### Pathogenicity in murine model

BALB/c mice with normal intestinal microbiota of 21 days and an average weight of 10 g were orally inoculated with 100 μl PBS containing 10^9^ bacteria [[41]. Five or ten mice were used per strain for this assay. The animals were housed in filtered air (HEPA filters) individual cages, with free access to food and sterile water. Survival and weight were recorded daily and morbidity was analyzed in duplicate by quantifying serum urea (g/l) on day three post-inoculation. Mice inoculated with PBS or *E*. *coli* DH5α were included as negative controls. Histological alterations in cecum and colon were analyzed by optical microscopy (magnification 400x). Statistical analyses were performed on uremia values by one-way ANOVA with the Tukey test for multiple comparisons. Lethality was analyzed using the Mantel-Cox test. The tests were conducted with the approval of Comite Institucional para el cuidado y uso de animales de experimentación (Institutional Committee for care and handling of experimental animals, CICUAE) of the Center for Veterinary and Agronomical Research (CICVyA) of Instituto Nacional de Tecnología Agropecuaria (INTA), Argentina (http://inta.gob.ar/documentos/cicuae-comite-institucional-para-el-cuidado-y-uso-de-animales-de-experimentacion). All animals were euthanized by CO_2_ inhalation before the experimental endpoint. We established the human endpoint when mice met one of the following signs: Loss of > 20% of the initial weight, lethargy, bristling coat, hemorrhagic diarrhea, and cry or howl when touched. Sixty-seven percent (n = 10) of animals were euthanized due to weight loss, 20% (n = 3) due to lethargy and 13% (n = 2) due to lethargy and weight loss

## Results

### SNP typing

**Fig 2:**
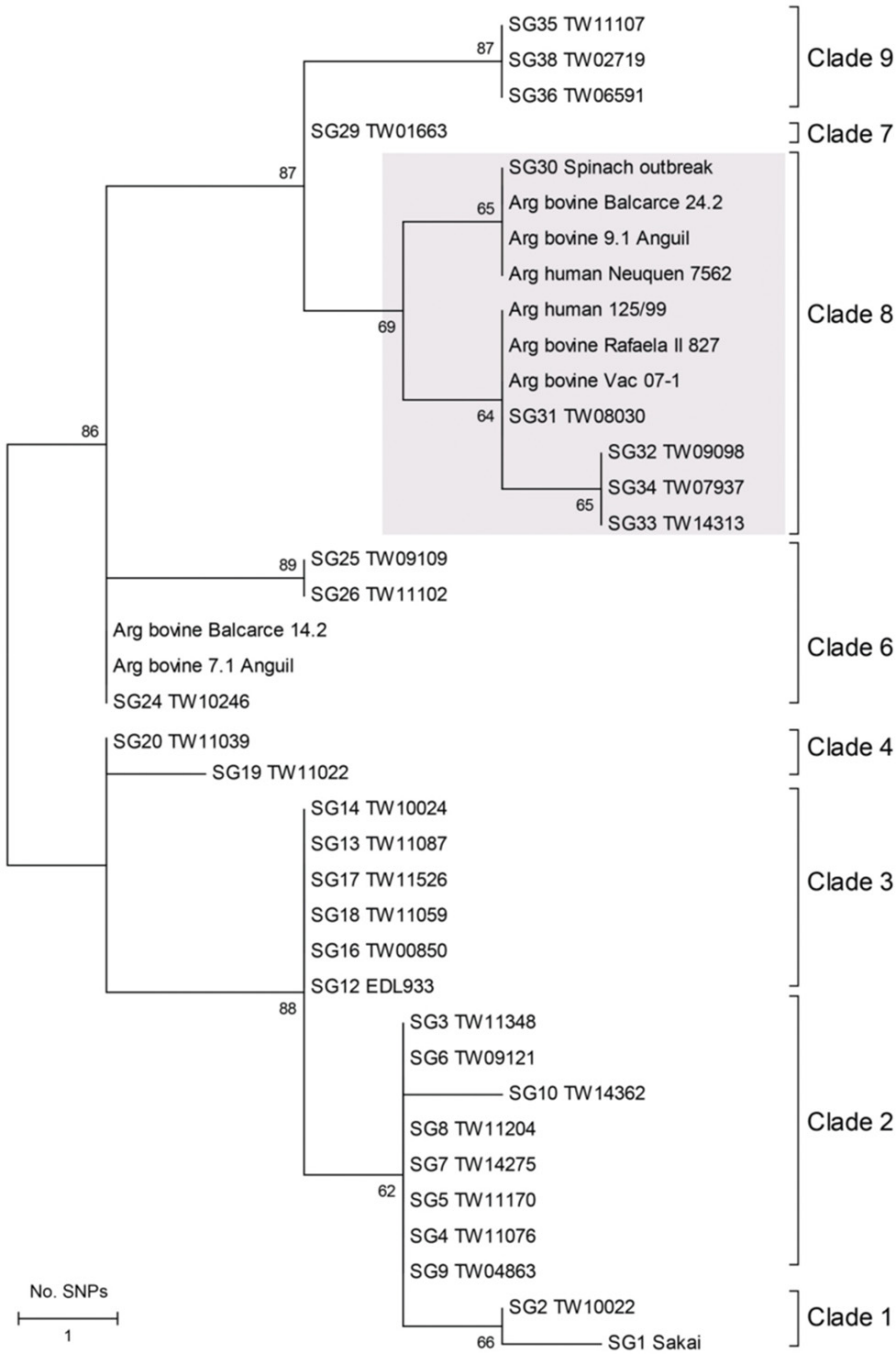
Neighbor-joining phylogeny of bovine-derived *E*. *coli* O157:H7 strains from Argentina and a subset of O157:H7 control strains representing single nucleotide polymorphism (SNP) genotypes (SGs) from eight of the nine previously defined clades [15]. The phylogeny was constructed using 23 SNP loci. The numbers at the nodes represent the bootstrap support following 1,000 replications, while strains belonging to clade 8 are indicated by the gray box.

SNP typing was used to genotype the strains with two methods. One proposed by Riordan et al [28] using 4 SNPs and another based on 23 SNPs that were shown to differentiate between the nine previously described clades [15]. With the method by Riordan et al. (28), six bovine isolates (7.1 Anguil, 9.1 Anguil, Vac 07–1, Rafaela II- 827, Balcarce 14.2, Balcarce 24.2) did not fit into any of the known clades, whereas two bovine strains (146N, 438/99) were classified as clade 3 (Table 1). The two local human strains were also unclassifiable using this method, which may be due to the low number of SNPs evaluated. In contrast to these results, with the method based on 23 SNPs, four of the six bovine isolates and both human isolates were classified as clade 8 in the Neighbor-Joining phylogeny (Fig 2). Specifically, one bovine and one human strain each clustered with control strains representing SNP genotypes (SG) 30 and 31 of clade 8. By contrast, the two bovine strains, 7.1 Anguil and Balcarce 14.2, had SNP profiles that clustered together with control strains belonging to clade 6 (Fig 2). In consequence, the 23 SNP based method allows a stronger assignment of clades as it uses more loci.

### Stx typing

The strains were characterized by the stx type and variants of the stx2. All the cattle strains harbor *stx2* but not *stx1* and six out of eight strains had two copies of *stx2* composed of the *stx*_*2a*_ and *stx*_*2c*_ variants.

Concerning the occupancy of classical insertion sites of Stx*2* phages *E*. *coli* O157:H7 146N, 125/99, 9.1 Anguil, Balcarce 14.2 and Neuquén 7562 had these insertions in *yehV*. For the other strains, however, the insertion site was not identified, though *wrbA*, *argW* or *sbcB* remained intact (Table 1 ND*: not determined). Importantly, this method does not detect an insertion of the entire *stx* phage, but it detects any insertion with a phage that may or may not contain a *stx* gene.

### Stx2 activity

**Fig 3:**
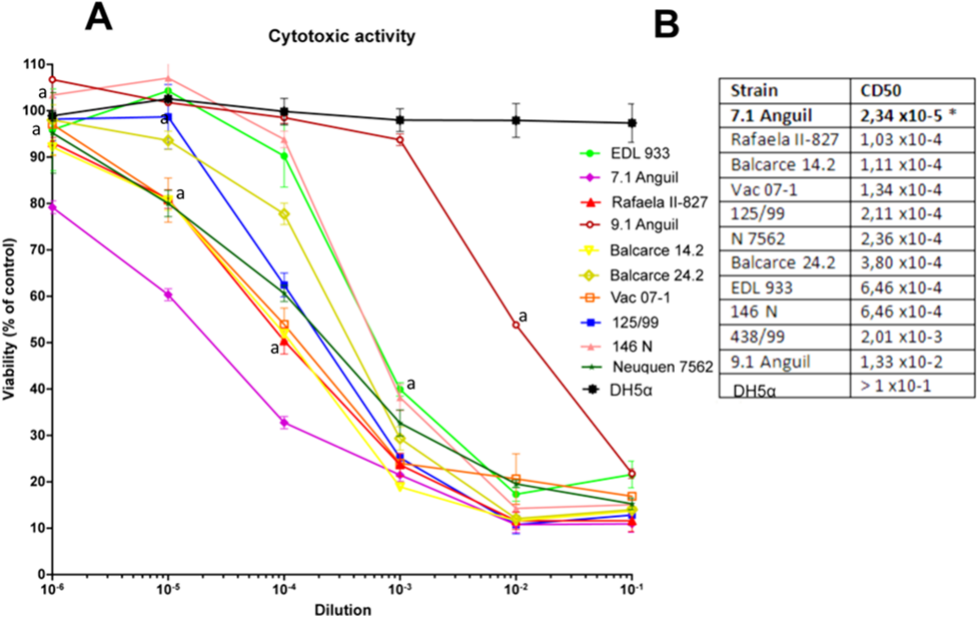
Cytotoxic activity (dilutions 10−^1^ to 10−^6^) of culture supernatant isolates of *E*. *coli* O157: H7 in Vero cells (A) and CD50 table (B). A:-induced cytotoxicity was observed with the analyzed supernatants at different dilutions. The results are expressed as percentage of detected neutral red (± 1 SEM); a 100% represents cells incubated without supernatant (control viability). DH5α strain represents a control devoided of Stx activity. Each assay was performed in triplicate, whereas each viability control was assessed by sextuplicate. a letters denoted statistically significant differences (p< 0.01) between the cytotoxic activity of *E*. *coli* O157:H7 7.1 Anguil and the rest of the strains in given dilution point. Only the closest point is marked as the other strains with lower activity has as expected also significantly lower activity B: CD50. The results are expressed as the required dose value of culture supernatant to present a Vero cell cytotoxicity of 50%. 100% represents cells incubated without supernatant (control viability). * = P <0.05.

As a correlate of virulence, we measured the Shiga toxin activity in bacterial culture supernatants in bovine-derived clade 8 isolates and in the two clade 6 strains (7.1 Anguil and Balcarce 14.2). Stx2 activity was measured in Vero cells (Fig 3). The CD_50_ was from 2.34 x10^5^ (strain 7.1 Anguil, bovine, clade 6) to 1.33 x10^2^ (strain 9.1 Anguil). The strain 7.1 Anguil presented a significantly higher level of activity (p<0.05) compared to all other strains. The standard EDL933 strain produced a CD_50_ of 6.46 x10^4^.

We also evaluated, semiquantitatively, the toxin released in the culture medium by ELISA [34]. Strain Vac 07–1 (bovine, clade 8) produced around double the amount of Stx proteins compared to the other strains, When mitomycin C was added the induction effect was high for Rafaela II-827 Vac 07–1, 7.1 Anguil, Balcarce 24.2 (approximately ten-fold higher) and notably higher for Balcarce 14.2 (24 times). (S1 Fig). It is important to note the cytotoxic activity in Vero cells (Fig 3) was performed with bacteria not treated with mitomycin.

In general there was agreement between the levels of verocytotoxic activity and the Stx production. A clear relationship between *stx2* copy number, and verocytotoxic activity in Vero cells or Stx production detected by ELISA could not be established. However. both assays showed that the top ranked isolates belongs to clade 8 or 6.

### Adherence to epithelial cell lines

**Fig 4:**
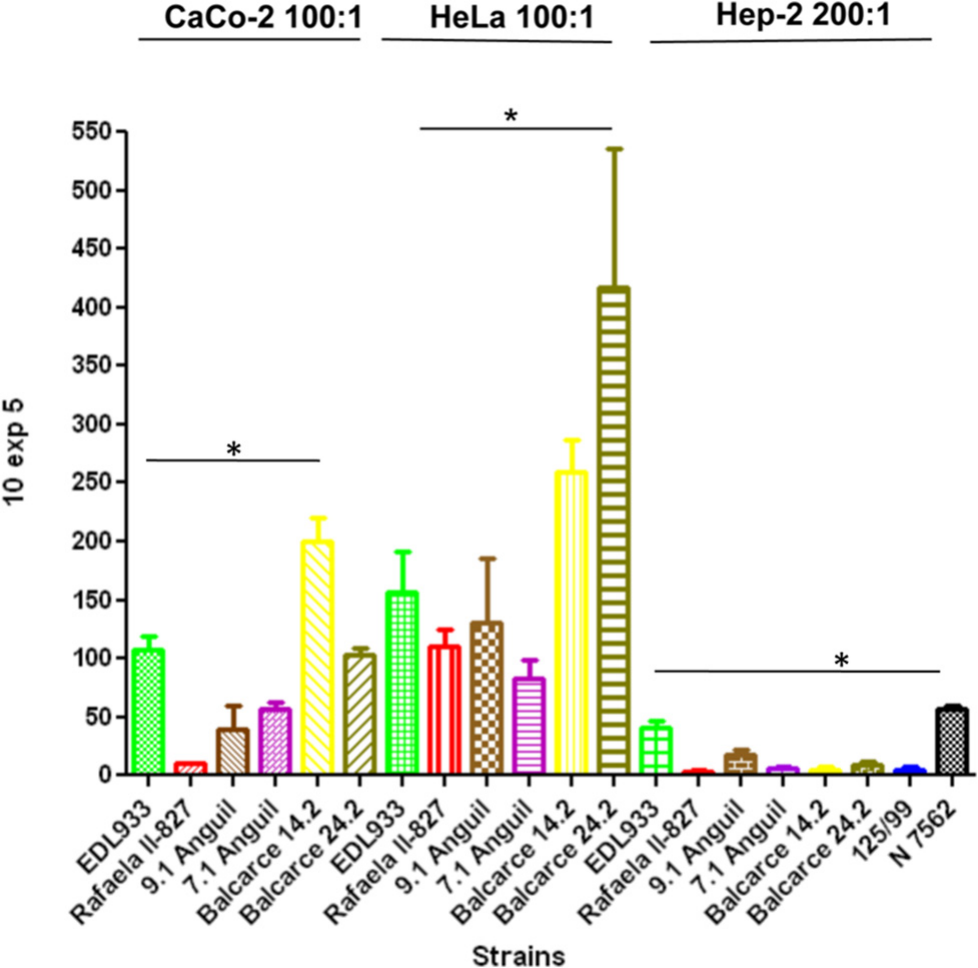
Adhesion of *E*. *coli* O157:H7 isolates to epithelial cells Caco-2, MOI:100; HeLa, MOI:100 and Hep-2 cells, MOI:200. Each assay was performed in triplicate. * = p <0.05.

Adherence to intestinal epithelial cells is a crucial step in EHEC infection. Three epithelial cell lines were selected for the adherence studies: HeLa (human cervix adenocarcinoma), HEp-2 (human larynx carcinoma) and CaCo-2 (human colorectal adenocarcinoma). HeLa cells promoted the highest adherence, with Balcarce 24.2 (bovine, clade 8) and Balcarce 14.2 (bovine, clade 6) showing the highest scores (p<0.05) followed by EDL933 (Balcarce 24.2 vs EDL933, p < 0.05). The strain Balcarce 14.2 was the most adherent to CaCo-2 cells (p<0.05), followed by Balcarce 24.2.and EDL933. The strain 7.1 Anguil, 9.1 Anguil and Rafaela II-827 were the less adherent strains (in decreasing order) with CaCo2 cells. HEp-2 was the cell line with the lowest adherence to bacteria (Fig 4). A qualitative analysis based on micrographs showed (S2 Fig) that the adherence was localized (LA) or localized-like type (LAL) [42].

### T3SS hemolytic activity

**Fig 5:**
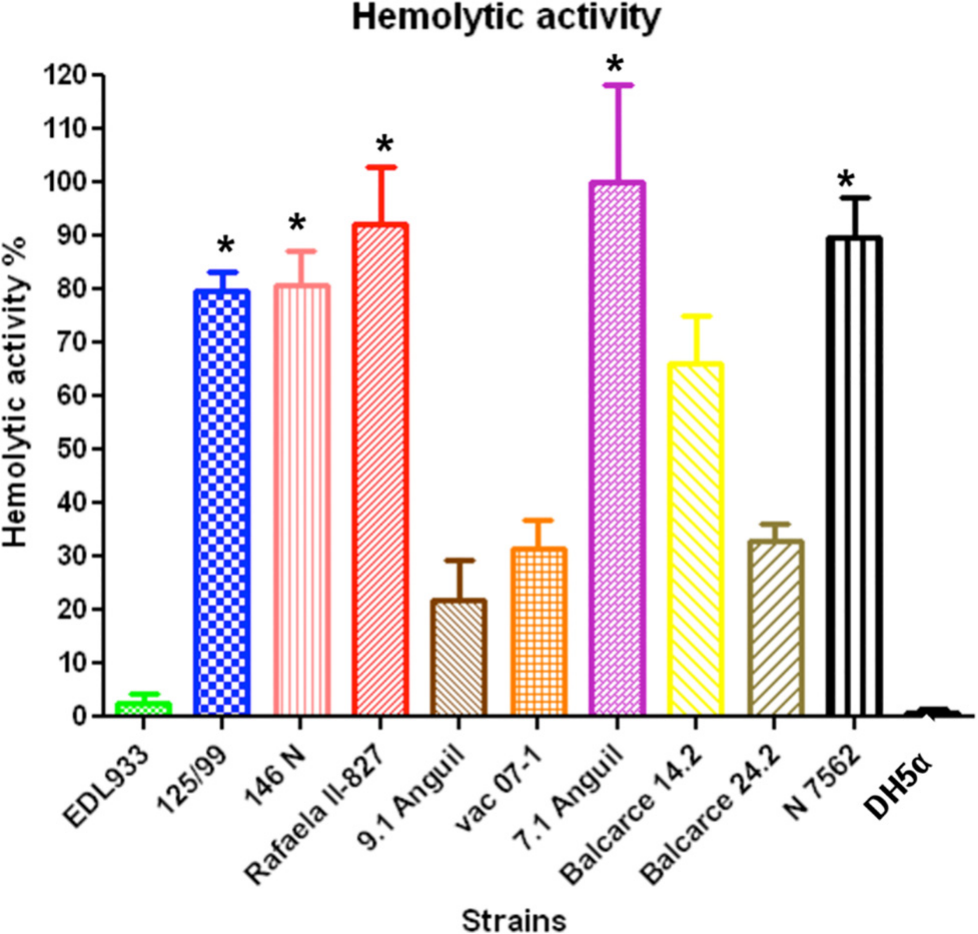
Hemolytic activity provoked by T3SS in of *E*. *coli* O157:H7 isolates from cattle from Argentina. Sheep erythrocyte hemolysis was observed with a significant (* = P <0.05) lysis induced by strains 7.1 Anguil, Rafaela II-827, Neuquen 7562, 146N and 125/99 in relation to the other tested strains. The results are shown as the relative percentage of hemolysis (± 1 SEM) in relation to the strain with the highest values (7.1 Anguil). DH5α represents hemolysis caused by a non-pathogenic strain. Each assay was performed in triplicate.

The RBC hemolysis caused by the T3SS is a correlate of T3SS physiological activity. For this reason, we performed hemolysis analyses. The cattle strain 7.1 Anguil was the most hemolytic and it was standardized to 100% hemolysis in ovine RBCs (Fig 5). Five of the isolates, 7.1 Anguil (bovine, clade 6), Rafaela II-827 (bovine, clade 8) and 146N, N7562 (human, clade 8) and 125/99 (human, clade 8), showed a high hemolytic activity (p <0.05) with values 80% higher than the other strains (Fig 5). EDL933 had a low activity, whereas the nonpathogenic DH5α strain that lacks a T3SS had no hemolytic activity. Supernatant of the bacterial isolates incubated in DMEM was then incubated with RBCs to verify if secreted hemolytic factors were present. Results were negative.

### Functionality evaluation of 7.1 Anguil strain in the human colon *ex vivo*

To observe the diarrheagenic potential of 7.1 Anguil (cattle, clade 6) with the highest Stx2 activity, we designed a functional test that allows the evaluation of 7.1 Anguil regarding to its capacity to modify fluid absorption across human colonic mucosa *in vitro*. This strain was compared to the standard strain EDL933. This experimental approach, previously employed to characterize the production of Stx2 in *E*. *coli* O157:H7 strain 125/99 [43] allows the simultaneous recording of the net water movement, electrical potential difference and short-circuit current across the intestinal barrier.

**Fig 6:**
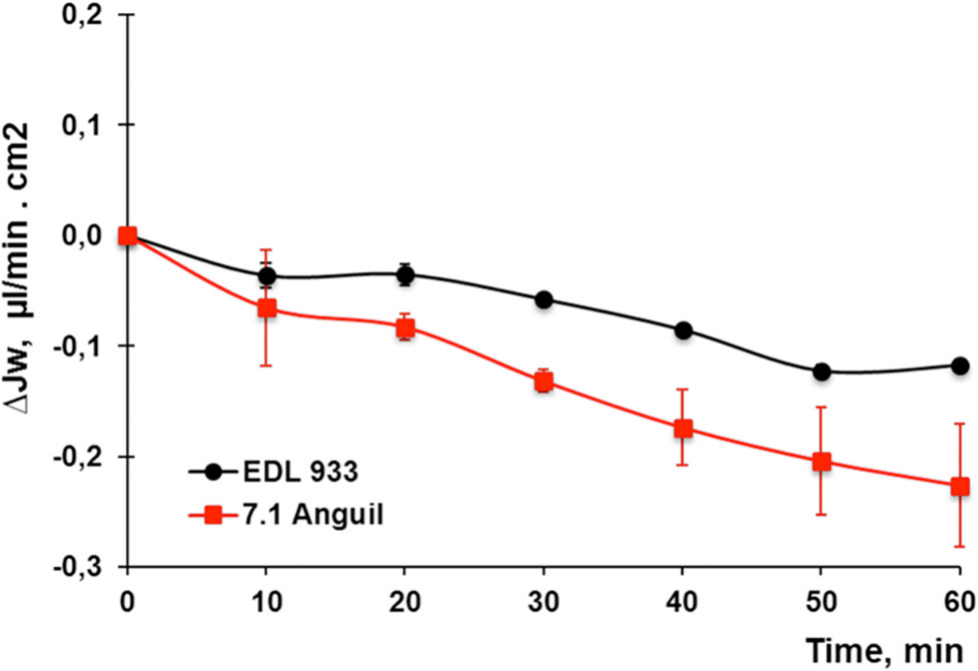
Effect of *E*. *coli* EDL933 and *E*. *coli* 7.1 Anguil strains on water absorption (Jw). Fragments of human colon were incubated for 60 minutes. The results are expressed as mean ± SEM (n = 3 for each experimental situation).

Absorptive Jw (0.24 ± 0.04 μl/min.cm2, n = 6) was observed in the human colonic mucosa placed between two identical Ringer solutions (2 ml each) in the Ussing chamber before the addition of bacteria. The electrical parameters tested simultaneously with water fluxes showed a PD of 1.6 ± 0.4 mV, Isc of 29.1 ± 4.2 μA/cm^2^ and Rt of 52.5 ± 11.9 Ω.cm^2^. Then, matched colonic mucosal obtained from the same patient were incubated with 200 μl (approximately 10^8^ CFU) of bacterial culture of the 7.1 Anguil or EDL933 strains. A significant inhibition of Jw was observed with both strains, although the level of inhibition was higher with 7.1 Anguil (Fig 6, p < 0.05, n = 3). Isc simultaneously measured in colonic mucosa incubated with 7.1 Anguil or EDL933 remained unchanged for at least for 60 min (**∆**Isc (μAmp/cm^2^): 5.1 ± 8.0 *vs* 1.0 ± 3.0, respectively, NS, n = 3). This functional test indicates the high capacity of 7.1 Anguil to cause inhibition of the normal water absorption in human intestine *ex vivo*.

### Pathogenicity in a murine model

A mouse model of EHEC infection that reproduces some of the clinical observations of HUS (death, weight loss, and renal failure) was used [44] to evaluate pathogenicty of the EHEC strains. In the first test (five BALB/c mice per group), animals were inoculated with all strains studied here: bovine clade 8 isolates (Rafaela II- 827 and 9.1 Anguil), clade 6 bovine isolates (7.1 Anguil and Balcarce 14.2) and the standard EDL933 strain. As controls, one group was inoculated with *E*. *coli* DH5α and the other with PBS. Animals inoculated with 7.1 Anguil suffered an elevated uremia (1.1 g/l) compared to to EDL933 (0.7), DH5α (0.6 g/l), Rafaela II-827 (0.6 g/l), 9.1 Anguil (0.5 g/l), Balcarce 14.2 (0.5 g/l), and the negative control (PBS, 0.5 g/l) (p values <0.05). The strain 7.1 Anguil showed a lethality rate of 60%, whereas Rafaela II-827 showed a rate of 20% (S3 Fig). No lethality was observed with all of the other strains or with PBS. Animals inoculated with *E*. *coli* O157:H7 7.1 Anguil, Rafaela II-827 and 9.1 Anguil suffered weight loss in descendent order (S3 Fig).

**Fig 7:**
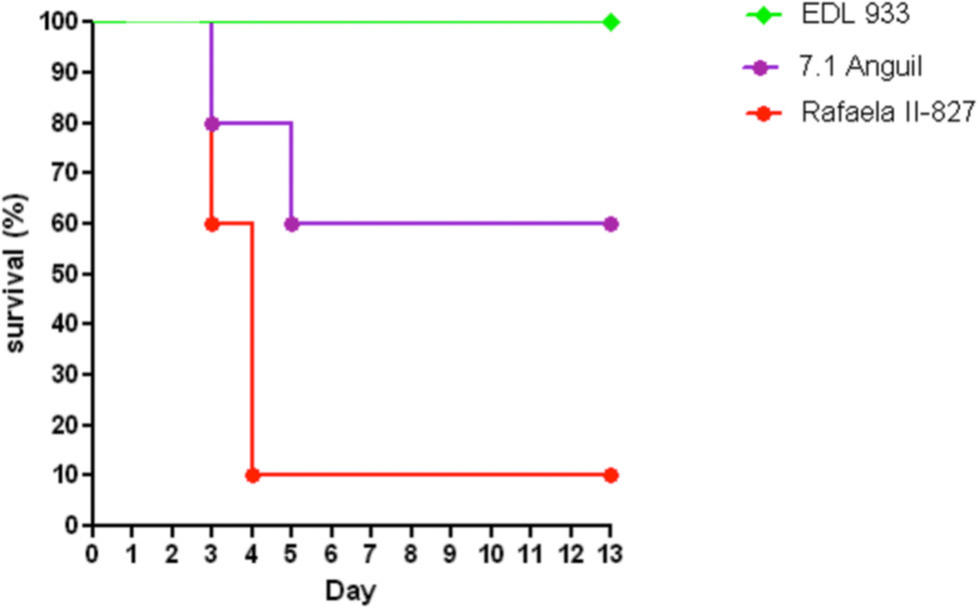
Mice infection with *E*. *coli* O157:H7 isolates. **Animals were intragastrically infected with *E*. *coli* O157:H7 at inocula of 10^9^ CFU in 200ul of PBS**. Survival after intragastric inoculation of BALBc mice with EDL933, 7.1 Anguil, 9.1 Anguil, Rafaela II-827, Balcarce 14.2, Balcarce 24.2, DH5α or PBS.

In a second test, Rafaela II-827 (clade 8, bovine), 7.1 Anguil (clade 6, bovine) and EDL933 were compared using larger groups of mice (10). A lethality rate of 90% was observed in mice inoculated with Rafaela II-827 (p<0.01), whereas a 20% rate was observed for 7.1 Anguil (Fig 7). A significant weight loss (p<0.05) was also observed in mice inoculated with Rafaela II-827 on day 4 post inoculation (data not shown). Importantly, the histological examination of mice infected with both Rafaela II-827 and 7.1 Anguil strains demonstrated renal necrosis at both glomerular and tubular level and microhemorrhagic foci. In the cecum, a marked mucosal destruction with loss of surface epithelium was also observed (S4 Fig).

## Discussion

Argentina has the highest incidence of HUS in the world [3,4,45,46]; however, the factors that contribute to increased incidence in this region have not been clearly identified. It should be noted that cattle are the main reservoir of EHEC and that Argentina has more cattle than people (over 55 million cattle and 42 million inhabitants). In a prevalence study, Mellor et al [25] identified 72% of clade 8 *E*. *coli* O157:H7 isolates in Argentina and only 2% in Australia in a sample from 120 animals. Fifty percent of isolates from Argentinean cattle were clade 8. The isolates examined in the current study came from Pampa of Argentine, where the majority of cattle are concentrated [47], and originated from four different geographical locations.

Cattle does not suffer from the disease caused by *E*. *coli* O157:H7 and for this reason no clinical records are available to associate with different genotypes of EHEC. In addition, no *bona-fide* animal model is known to reproduce the infection pattern of EHEC and the clinical signs of HUS in humans, which makes the comparison difficult. We therefore sought to study the potential virulence of clade 8 isolates from Argentinean cattle through the use of biochemical *in vitro* correlates of virulence and a mouse infection model.

Although the relative virulence of the *E*. *coli* O157:H7 isolates, both from humans and cattle, has been previously analyzed, few studies have examined whether phylogenetic lineage, or clade assignment impacts virulence *in vivo*. Baker et al [48], for instance, compared the virulence of strains isolated from cattle and humans in gnotobiotic piglets and observed that those from humans were more virulent to mice. Eaton et al [49] studied the kidney pathogenesis of different human EHEC strains in mice. In turn Shringi et al [50] also compared the virulence of strains isolated from cattle and humans in a piglet and rabbit model and observed that the human strains were more virulent than the bovine strains. However these studies have not determined if lineage impacts *E*. *coli* O157:H7 virulence. Strains from clade 6and clade 8 tend to produce higher levels of Stx2 toxin. A high production of Stx2 by clade 8 strains has been previously reported [17] along with the ability of Stx2 to alter the normal water absorption across human colon in a dose-dependent manner without altering the electric parameters [51]. In consequence, the higher inhibition of water absorption caused by 7.1 Anguil compared to EDL933 may be attributed to a higher production of Stx2 by 7.1 Anguil. Although a reduced number of isolates were reported here, studies of more isolates from clade 8 and 6 are in progress to more strictly assess the hyprevirulence characteristics of these genotypes.

This study did not show a clear association between *E*. *coli* O157:H7 lineages and hemolysis. More isolates of different lineages should be studied to draw a definitive conclusion. The epithelial cell adherence assay was more conclusive in HeLa cells and a clade 8 isolate and a bovine isolate belonging to a novel lineage were the most adherent strains to this type of cell, although other clade 8 strains were less adherent than *E*. *coli* O157:H7 EDL933. Adherence of *E*. *coli* O157:H7 to epithelial and abiotic surfaces is a complex process. More than 10 tested or putative adhesins are described and those studied have been recently reviewed by McWilliams and Torres [52]. We have observed (unpublished observations) that there is no polymorphism in the structural genes of the adhesins described McWilliams and Torres [52] between *E*. *coli* TW14359 (a prototypic clade 8 strain) and EDL933 and Sakai strain. The differences in adherence may be attributed to differential expression patterns among the strains studied here or to other recently described putative adhesins [53] that may be involved in adherence to these cells. Other authors also have observed differences in adherence to different surfaces in spite of similar adhesin composition among *E*. *coli* O157:H7 strains.[54].

The behavior of Argentinean strains in this study were not uniform among strains belonging to clade 8. A similar finding was observed in previous studies [22]. Importantly, the clade 8 Rafaela II-827 strain and the 7.1 Anguil strain, which was most closely related to strains of clade 6, were the most lethal and pathogenic for mice. These data suggest that two strains recovered from Argentinean cattle with distinct genetic backgrounds may have an enhanced ability to cause disease in humans. Nonetheless, these strains were not evaluated by the complete set of 96 SNP loci [15] and not all of the original SNP genotypes were included in the current study. As a result, additional genomic characterization of each strain is necessary to better understand the evolutionary history and identify factors that are most important for virulence and disease severity.

Further investigations are in progress to evaluate the genetic basis of virulence that contributes to variation by clade as well as the assessment of the ecological differences that contribute to variation in transmission rates and linkage to food and waterborne disease.

## Supporting Information

S1 Fig ELISA for detection of Stx in supernatant cultures of *E*. *coli* O157:H7. Supernatant was diluted 1:100 to have linear OD450 reads.
(TIF)

S2 Fig Adhesion of *E*. *coli* O157:H7 isolates to Caco-2 cells. All observations were performed under a microscope. A: Adherence to Caco-2 cells 7.1 Anguil strain, MOI 200. B: Adhesion to Caco-2 cells of Balcarce 14.2 strain, MOI 200. C: Adherence to Caco-2 cells of strain EDL933 (positive control), MOI 200. D: Adherence to Caco-2 cells of DH5a strain (negative control), MOI 200. The monolayers were fixed and stained with 10% Giemsa stain.
(TIF)

S3 Fig Mice infection with *E*. *coli* O157:H7 isolates. Animals were intragastrically infected with *E*. *coli* O157:H7 at inocula of 10^9^ CFU in 200ul of PBS. A. survival after intragastric inoculation of BALBc mice with EDL933, 7.1 Anguil, 9.1 Anguil, Rafaela II-827, Balcarce 14.2, Balcarce 24.2, DH5α or PBS. B. evolution of the weight of the mice before and after *E*. *coli* O157:H7 inoculation.
(TIF)

S4 Fig Mice histopathology. Kidney sections (A, C, E) and cecal mucosa sections (B, D, F) of BALB/c mouse infected with control strain DH5α (A, B), O157:H7 strain 7.1 Anguil (C, D) or with O157:H7 Rafaela II-827 (E, F). The mouse was necropsied on day 3 or 4 post-infection. Both strains, 7.1 Anguil and Rafaela II-827, caused glomerular and tubular renal necrosis level (arrows) and microhemorragc foci; in cecum mucosal destruction and necrosis was observed (triangles), with epithelial suface loss and inflammatory infiltration (asterisk). H&E stainning, 400x magnification.
(TIF)

S1 Table SNP loci evaluated. (XLSX)
