# Supplementary figures and images for: Clade 8 and Clade 6 Strains of Escherichia coli O157:H7 from Cattle in Argentina have Hypervirulent-Like Phenotypes
Source: PLoS One. 2015 Jun 1;10(6):e0127710. doi: 10.1371/journal.pone.0127710 (PMC4452545; doi:10.1371/journal.pone.0127710)

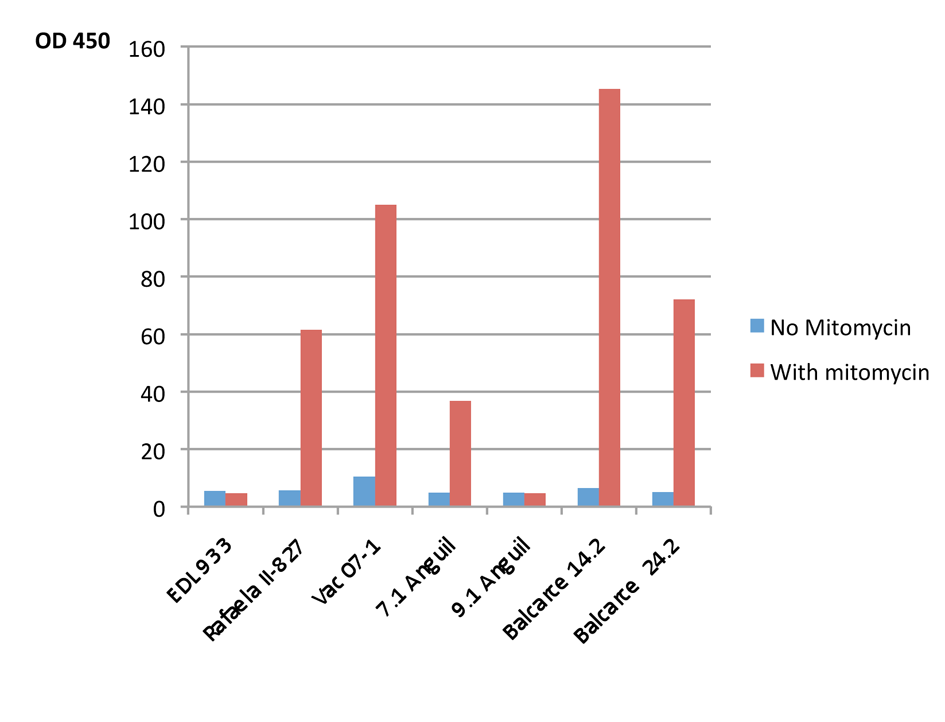

Supplement: S1 Fig — Supernatant was diluted 1:100 to have linear OD450 reads. (TIF) [file pone.0127710.s001.tif]

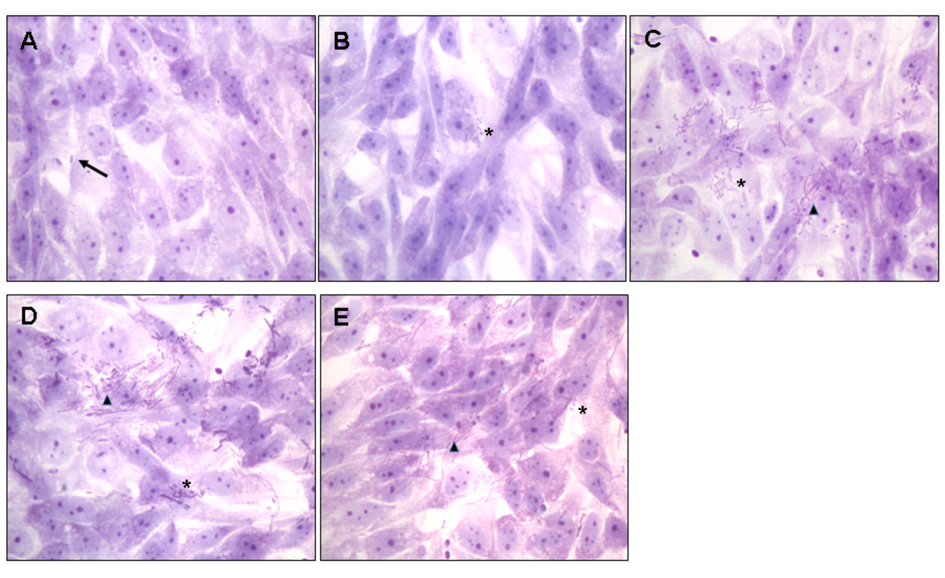

Supplement: S2 Fig — All observations were performed under a microscope. A: Adherence to Caco-2 cells 7.1 Anguil strain, MOI 200. B: Adhesion to Caco-2 cells of Balcarce 14.2 strain, MOI 200. C: Adherence to Caco-2 cells of strain EDL933 (positive control), MOI 200. D: Adherence to Caco-2 cells of DH5a strain (negative control), MOI 200. The monolayers were fixed and stained with 10% Giemsa stain. (TIF) [file pone.0127710.s002.tif]

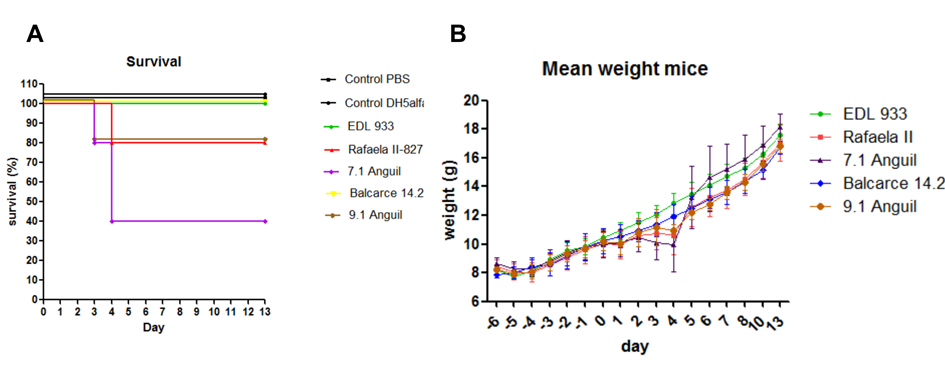

Supplement: S3 Fig — Animals were intragastrically infected with E. coli O157:H7 at inocula of 109 CFU in 200ul of PBS. A. survival after intragastric inoculation of BALBc mice with EDL933, 7.1 Anguil, 9.1 Anguil, Rafaela II-827, Balcarce 14.2, Balcarce 24.2, DH5α or PBS. B. evolution of the weight of the mice before and after E. coli O157:H7 inoculation. (TIF) [file pone.0127710.s003.tif]

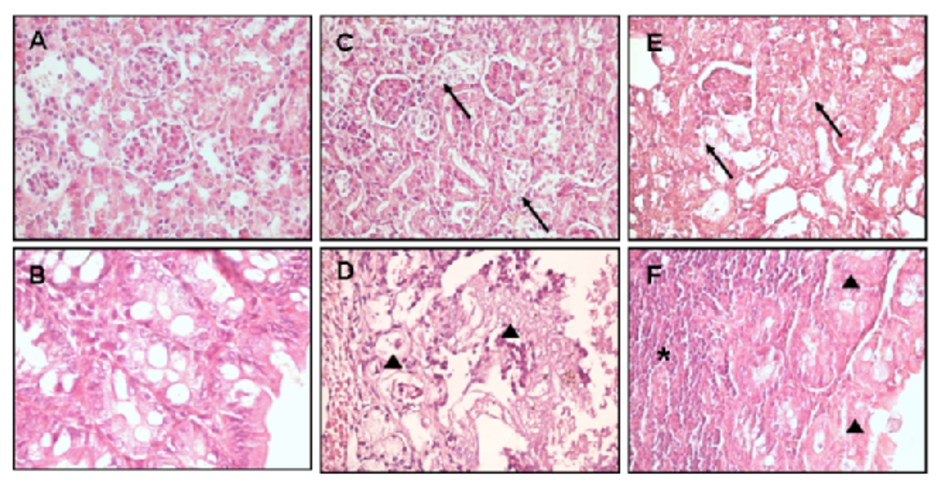

Supplement: S4 Fig — Kidney sections (A, C, E) and cecal mucosa sections (B, D, F) of BALB/c mouse infected with control strain DH5α (A, B), O157:H7 strain 7.1 Anguil (C, D) or with O157:H7 Rafaela II-827 (E, F). The mouse was necropsied on day 3 or 4 post-infection. Both strains, 7.1 Anguil and Rafaela II-827, caused glomerular and tubular renal necrosis level (arrows) and microhemorragc foci; in cecum mucosal destruction and necrosis was observed (triangles), with epithelial suface loss and inflammatory infiltration (asterisk). H&E stainning, 400x magnification. (TIF) [file pone.0127710.s004.tif]
